# Fall prevention indicator priorities for public health and across health sectors in Ontario: a comparative study

**DOI:** 10.1186/s12877-025-05693-3

**Published:** 2025-11-03

**Authors:** Sarah A. Richmond, Alexia Medeiros, Ian Pike, Megan Oakey, Alison K. Macpherson

**Affiliations:** 1 Health Promotion, Chronic Disease & Injury Prevention, Public Health Ontario, 661 University Ave, Toronto, ON Canada; 2 Dalla Lana School of Public Health, University of Toronto, Toronto, ON Canada; 3 British Columbia Injury Research and Prevention Unit, BC Children’s Hospital Research Institute, Vancouver, BC Canada; 4 Department of Pediatrics, The University of British Columbia, Vancouver, BC Canada; 5 BC Centre for Disease Control, Provincial Health Services Authority, Vancouver, BC Canada; 6 School of Kinesiology & Health Science, Faculty of Health, York University, Toronto, ON Canada

**Keywords:** Indicators, Injury prevention, Accidental falls, Policy, Wounds and Injuries

## Abstract

**Introduction:**

Falls are the leading cause of injury-related emergency department (ED) visits and hospital admissions among older adults across many provinces in Canada. To effectively address this burden requires relevant data and indicators to inform fall prevention planning and evaluation for practitioners across the spectrum of prevention.

**Methods:**

We used a modified Delphi approach, including an environmental scan, survey and pairwise comparison exercise to identify, refine and prioritize older adult fall prevention indicators across multiple health sectors in Ontario and specifically for public health. Three iterative phases of consultation were conducted with practitioners, as well as experts in injury prevention indicator development.

**Results:**

The prioritization exercise resulted in differing priorities between multiple sectors and public health. The highest ranked indicator for multiple sectors was *the rate of ED visits,* and the lowest was *disability-adjusted life years due to a fall.* For public health, *the rate of hospitalizations due to a fall* was ranked first, with *the rate of mortality due to a fall* last. The remainder of the list differs considerably by group, with certain indicators ranked on one list, but not the other.

**Conclusion:**

This work identified, refined and prioritized indicators for older adult fall prevention across health sectors and for public health in Ontario. While both groups shared some highly ranked indicators, their differing responsibilities in fall prevention are reflected in the contents and order of their respective priorities for indicators. Delineating the unique data needs of each group highlights the importance of having consistent and actionable data that informs prevention planning and evaluation.

**Supplementary Information:**

The online version contains supplementary material available at 10.1186/s12877-025-05693-3.

## Introduction

In Canada, falls are the leading cause of injury-related hospital admissions among older adults (ages 65 +) representing approximately 87% of all injury hospitalizations in this population [1]. Further, 20–30% of older adults experience a fall each year, in Canada [2]. The burden of falls is similar across Canadian provinces, including Ontario, where in 2021 there were 146,203 emergency department (ED) visits for fall-related injuries among older adults [3]; 36,530 were subsequently hospitalized [4]. Falls among older adults are likely to cause injuries such as hip fractures [5]—a significant injury for older adults which typically results in hospitalization [6]. What places an older adult at risk of a fall is influenced by a wide range of biological, behavioral, socio-economic and environmental factors [2]. As a result, the responsibility of preventing falls is shared across sectors and includes healthcare providers, researchers, multiple levels of government and public health practitioners [2].

Opportunities for fall prevention among older adults exist across the health-related continuum [7]. This includes universal primary prevention efforts for those at low risk of a fall, usually intended for community-dwelling older adults, to targeted secondary prevention for those at higher risk, often in residential care [8]. For example, public health practitioners often collaborate with local organizations to implement primary prevention programs for community dwelling older adults using a proportionate universalism approach [9]. This includes exercise-based training programs to increase lower extremity strength and balance [10]. Practitioners in long-term care with high-risk older adults would assess falls risk, and implement targeted individual prevention strategies based on multiple risk factors [10]. Individual sectors may play a greater or lesser role across the continuum; however, it is acknowledged that across sectors, an evidence-informed approach to developing, implementing and evaluating fall prevention programs is necessary [9]. This approach requires the use of necessary data, evidence, and relevant information across all stages of program development.

In Ontario, the Ontario Fall Prevention Collaborative (OFPC) was developed to address falls among older adults, supporting and endorsing a multi-sector approach to prevention [11]. Members include practitioners from across the province, consisting of a variety of health professionals representing a number of sectors, including acute care, rehabilitation care, home and community care, and public health. This group acknowledged a gap in their approach including a lack of specific and actionable data and indicators [11]. It was determined that indicators were needed that could be used across all health sectors to: 1) understand the true incidence of falls, 2) develop appropriate fall prevention interventions using known risk and protective factors, and 3) implement and evaluate the effectiveness of fall prevention programming. It was hypothesized that the use of a set of indicators to monitor the fall prevention system would provide the opportunity to compare data across sectors and geography, track trends over time, and ensure consistent reporting on the effects of programs to prevent falls among older adults. It was also hypothesized that the indicators that are used across the system may not be different to those in public health practice.

Public health plays an upstream role in fall prevention. Distal to clinical care but proximal to population level intervention, public health provides fall prevention programming primarily for community-dwelling older adults in collaboration with local organizations such as recreation, community care and family health teams. Previous work set to understand current challenges and needs for public health practice in Ontario determined that access to local data and indicators for injury prevention broadly, and for program areas such as fall prevention specifically, were needed to build fall prevention programs within public health [12]. Indicators in public health are primarily used in planning, in determining strategic priorities and, in some cases, program design; however, they are typically not used during implementation or evaluation work. Anecdotally, practitioners describe that outcome indicators such as ED visits, hospitalizations and mortality, which frequently make up public health indicators, are not suitable to evaluate specific programs. More importantly, practitioners describe the inability to demonstrate a causal effect in most population health efforts using existing fall indicators.

With an aging population and consistently high burden of falls in Canada and Ontario, it is necessary to utilize relevant indicators to support a coordinated effort across health sectors to prevent fall-related injuries. Currently, the only indicators consistently reported for community dwelling older adult falls include the rate of ED visits, hospitalizations and mortality [13]. While these outcome indicators are useful for capturing the population burden of falls, they are not sufficient for informing specific fall prevention programming or measuring the impact of fall prevention interventions. To inform the development and evaluation of fall prevention programming, it is necessary to have indicators that are relevant and actionable for use both across sectors and within specific sectors. As the need for knowledge provided by data varies by setting, it is imperative to understand which indicators hold the highest priority within each sector, and to understand how the value of various indicators may be similar or different by sector in order to inform and obtain the most useful data to populate and information for fall prevention work.

To meet this need, we sought to identify and prioritize indicators for fall prevention that inform work across multiple sectors in health and, as public health researchers, indicators specific to public health, and to compare the priorities of each group.

## Methods

A modified Delphi (mDelphi) approach, including an environmental scan, was employed to identify and prioritize older adult fall prevention indicators for health sectors across Ontario and those specific to public health. We used the Conducting and Reporting Delphi Studies (CREDES) guideline in reporting our results (Supplementary Table 1.). The environmental scan has been previously published [14]; the work reported here differentiated to include a comparison of indicators between public health and other health sectors. We used a mDelphi approach as our goal was to reach consensus among participants on a list of indicators, ranked by priority, for use in older adult fall prevention. This work did not include a priori consensus threshold nor a set number of rounds for reaching consensus as literature in this area suggests these limits may be arbitrary from both a conceptual and practical purview if indicators considered relevant fall below the a priori cut off [15]. This methodology has been used previously in priority-setting exercises for injury prevention [16] and indicator development [17–19]. We engaged participants identified through the OFPC to represent the multi-sector group, practitioners from public health for the public health group, as well as an expert advisory group (i.e., academics) with extensive experience in injury indicator development. The list of sectors represented in both the public health and the multi-sector group can be found in Table 1. Examples of the types of practitioners that represented each group included public health nurses and health promotion specialists in the public health group and clinic managers, elder life specialists, and program coordinators in the multi-sector group. Both the OFPC and our expert advisory group were consulted throughout the project, provided recommendations and re-oriented collaborative efforts toward project goals. Finalizing the list of indicators for both the multi-sector and public health groups was completed over three iterative phases and included multiple rounds of discussion. We completed this work first for the multi-sector group and then for public health. Primary research ethics approval for this project was received by the Ethics Review Board of Public Health Ontario (2019–051.01). All participants involved in this research completed consent forms.

**Table 1:** Sectors represented in multi-sector and public health participant group, phase two and three

Sector	Survey Participants	Reaching Consensus Participants
**Multi-Sector Group**		
Long Term Care	3	2
Rehabilitation	2	1
Home, Community Care	4	3
Not-for-Profit, Academic	3	3
Hospital, Acute Care	3	2
**Public Health Group**		
Public Health Practitioners	4	5

### Phase 1: indicator identification

Step one of this process was to identify indicators for older adult fall prevention that are currently in use in Canada and internationally. This involved conducting an environmental scan including a peer-reviewed literature search using relevant databases and search terms. The databases searched included the Cochrane Evidence Based Medicine Reviews, Medline (OVID), Cumulative Index to Nursing and Allied Health Literature (CINAHL EBSCO) and PsycInfo (APA). Search terms included “fall,” “prevention,” “seniors,” and “indicator” as well as related possible alternatives. We also included a grey literature search of 29 non-peer-reviewed sources including government and non-governmental reports, academic dissertations and conference presentations to identify all potential indicators relevant to older adult fall prevention. The results of this process have been previously described in more detail elsewhere [20]. Further, members of each group were asked to identify indicators that they currently use in practice, as well as recommendations. This included indicators that were not currently reflected in the list generated by the environmental scan, but were deemed important and that would prompt action in fall prevention. These indicators were added to supplement the results of the literature search. The results of these two steps were compiled into a fulsome candidate list of older adult fall prevention indicators.

### Phase 2: indicator prioritization

The list of candidate indicators identified in the environmental scan was presented back to participants in each group and our experts (i.e., academics in injury indicator development and prioritization) in order to obtain feedback on its contents. This process involved removing duplicate suggestions and refining recommendations such that candidate indicators had measurable numerators and denominators. These refined candidate indicators then formed the list to be used in next phase of the mDelphi process.

In Phase 2, we administered an online survey (using Surveys@PHO software) where participants were asked to score each indicator from the refined candidate list on three criteria: 1) usefulness (how useful the indicator is at informing decision-making), 2) feasibility (how feasible the indicator is to collect and report on), and 3) ability to prompt action (how likely the indicator is to prompt action in fall prevention). The selection of these criteria was informed by previous work in this area [16–18] and expert consultation. A Likert-type scale, scored from 1–9, was used to rate the indicators, where 1 did not satisfy the criterion and 9 satisfied the criterion extremely well. Participants were instructed to consider how these indicators met each criterion for fall prevention among older adults. For the multi-sector group, participants were instructed to rank indicators on how they met each criterion broadly, across health sectors and the public health group, specific to their sector or their own work. The average ranking of each indicator was calculated based on their scores for each criterion, and across the three criteria. The ranked list of indicators was used in the second round of prioritization.

A third-party qualitative research organization was hired to facilitate the second, online indicator priority-setting exercise (using Zoom™ Video Communications). First, the ranked list of indicators was presented back to participants from the first round of prioritization and placed into three categories based on their scores from the survey: High Priority, Medium Priority and Low Priority in order to enable discussion and final priority-setting. Scores from the survey ranged from 79–156 out of a possible 171 points for each individual criterion and 281–457 out of a possible 513 across the three criteria, as each participant (*n* = 19) could allot between 1 and 9 points for each indicator on each criterion. Participants were then asked to discuss the average ranking of each indicator within and between categories.

The final online indicator prioritization activity used a pairwise comparison method [16] that involved comparing each indicator against another. Given the large number of OFPC members, one participant was recruited to represent each sector for the multi-sector group in order to ensure that all perspectives were considered equally. We used the criterion ‘ability to prompt action’ for this exercise, based on expert opinion and previous work in this area [16]. The indicator that received the most votes in each comparison was awarded one point; the indicators were then ranked based on their total scores from the exercise for each group (i.e., multi-sector and public health).

### Phase 3: reaching consensus

The ranked list of indicators from the pairwise comparison exercise was presented back to participants for a final round of feedback, and to ensure that consensus was reached among all participants on the prioritized order of the indicators, and in particular, the contents of the top 10 indicators. The list was presented to each group, and discussion through the trained facilitator was conducted through an online discussion forum. We did not specify a priori, the number of rounds to achieve consensus, but consensus was considered reached when participants did not want to see any other changes to the list. Discussion was facilitated by asking participants to reference the ranked list and discuss any movement on the placement of indicators, if they felt the current placement did not reflect their view, as well as to provide their reasoning for any suggested changes. At this stage, the list was also discussed with the expert group for final feedback.

## Results

The results of the ranking exercises are presented in Table 2 as the top 10 indicators in each group. In reaching consensus, participants engaged in one round of discussion, resulting in the final list of indicators. The multi-sector group (*n* = 11) ranked *the rate of ED visits* highest and *disability-adjusted life years due to a fall* in 10th place. For public health (*n* = 5), *the rate of hospitalizations due to a fall* was ranked first, with *the proportion of dedicated fall prevention leads* last. Ranked second was *the direct and indirect health care costs related to falls* by the multi-sector group, and the *rate of ED visits* for public health. *Direct and indirect costs* were ranked 6th for public health and *hospitalizations* 5th for the multi-sector group. Other than the two existing indicators for public health (i.e., *ED visits* and *hospitalizations*), *the proportion of falls by place of occurrence* ranked highest. The remainder of the list differs significantly by group, with certain indicators ranked on one list, but not the other (e.g., *proportion of ambulatory calls transported vs. not transported, the proportion of older adults participating in strength and balance programs* and *the proportion of home risk assessments completed*). These indicators are presented in Table 3, as de-prioritized indicators, including an indication of which group(s) de-prioritized each listed indicator.

**Table 2:** Top 10 prioritized indicators, multi-sector vs. public health

	**Multi-Sector**	**Public Health**
**1.**	Rate of emergency department visits due to a fall	Rate of hospitalizations due to a fall
**2.**	Direct and indirect costs associated with fall-related injuries	Rate of emergency department visits due to a fall
**3. **	Proportion of dedicated fall prevention leads, at a system level	Proportion of falls by place of occurrence/activity associated with a fall
**4.**	Proportion of older adults with a comprehensive falls risk screening and assessment completed	Rate of serious fall-related injury hospitalizations
**5.**	Rate of hospitalizations due to a fall	Proportion of older adults with a comprehensive falls risk screening and assessment completed
**6.**	Rate of mortality due to a fall	Direct and indirect costs associated with fall-related injuries
**7.**	Proportion of ambulatory calls transported vs. not transported	Proportion of older adults participating in strength and balance programs
**8.**	Proportion of falls by place of occurrence/activity associated with a fall	Proportion of home risk assessments completed
**9.**	Rate of serious fall-related injury hospitalizations	Rate of mortality due to a fall
**10.**	Disability-adjusted life years due to a fall	Proportion of dedicated fall prevention leads, at a system level

**Table 3:** De-prioritized indicators, multi-sector and public health

Indicators	Multi-Sector	Public Health
Rate of repeat emergency department visits due to a fall		**X**
Average length of stay in hospital due to a fall	**X**	**X**
Rate of general health assessments completed	**X**	**X**
Rate of falls within hospital/long-term care	**X**	**X**
Disability-Adjusted Life Years (DALY's) due to a fall		**X**
Availability of education programs/resources for healthcare staff related to injury prevention	**X**	**X**
Proportion of older adults participating in strength and balance programs	**X**	
Proportion of home risk assessments completed	**X**	
Proportion of public health units that include fall prevention in their strategic plans/frameworks/have a dedicated fall prevention lead	**X**	**X**
Fall prevention score (assigned at a system level) based on actions taken toward fall prevention	**X**	**X**
Rate of transfer to increased level of care	**X**	**X**

There were no changes to the final presented list of indicators in Phase 3 as all participants communicated satisfaction with the final results of the pairwise comparison exercise.

## Discussion

Consensus on which indicators are important for older adult fall prevention in Ontario and their order of priority was reached for both the multi-sector and public health groups. The results of each group’s ranking varied significantly, highlighting the differing functions of the sectors represented in each group and therefore, the specific indicators that would benefit their work. Both groups ranked *the rate of ED visits due to a fall* high, likely reflecting the importance for both groups to understand and have the ability to present the population burden of fall-related injuries in their area. Typically, indicators are developed to provide monitoring and evaluation of a system. Future work including a continued process across other sectors could arrive at a parsimonious set of indicators that include multi-sector and public health as relevant players in the system supporting older adult falls prevention, however at this stage, there are still discrepancies in the priority indicators for each group.

Public health indicated the *proportion of falls by place of occurrence/activity* as their highest ranked indicator, after ED visit and hospitalization indicators. This is due to its use in identifying high risk places and activities that can guide public health intervention planning. Currently, the most widely collected and accessible administrative data sources that could be used to populate this indicator are the National Ambulatory Care Reporting System (NACRS) and the Discharge Abstract Database (DAD), that contain information on all ED visits and hospitalizations in Ontario. While these data sources include ICD-10 codes that describe the external cause of both intentional and unintentional injury (V00-Y33), as well as a code describing the place of occurrence of the injury (U98), these alone do not provide sufficient detail to inform intervention planning. For example, the range of external cause codes for falls (W00-W19) broadly categorizes the cause of the fall (e.g. W00- fall on the same level involving ice and snow), but provides no additional information on how the fall occurred. Similarly, the codes that describe where the injury occurred are broad and limited (e.g. U98.1 residential institution, U98.9 unspecified place of occurrence) and do not include any specific information on where within that location the injury occurred. While this information would provide public health units (PHUs) with more detail about the falls in their population than they currently have access to, there may be a need to access other data sources with more detailed place and activity information to maximize the utility of this indicator for users. For those in other sectors, this information may have been considered less critical.

The public health group ranked *the rate of hospitalizations due to a fall* in second place; ranked 5th by the multi-sector group. This may reflect the importance for public health to capture falls that result in more serious injuries and require hospitalization. This is additionally supported by the public health group’s high average ranking of the *rate of serious fall-related injury hospitalizations* indicator, as this indicator further distinguishes the most severe fall-related injuries, and is a more reliable reflection of serious injury than hospitalizations alone. Members of both groups had initially indicated their use of the indicator *length of stay in hospital due to fall-related injuries* as a proxy for injury severity; however, further discussions noted how the effects of existing comorbidities as well as the patient’s discharge status may confound the relationship between injury severity and the length of a hospital stay. This resulted in this indicator ultimately being de-prioritized (Table 3). The need for a more appropriate indicator to capture this construct was reported, and reflected in the high-average ranking of *the rate of serious fall related hospitalizations* for the public health group.

The importance of severity for both groups was further demonstrated in the discourse around the indicator *disability adjusted life years due to a fall*. Participants from hospital and acute care indicated that they had previously used this measure in an attempt to capture the severity of fall-related injuries; however, it was reported that this indicator does not sufficiently reflect the concept of severity for an older adult population over its use in a younger population. This is demonstrated in the indicator ranking lowest for multi-sector, and de-prioritized outside of the top 10, for public health.

Interestingly, both groups ranked *the proportion of older adults with a comprehensive falls risk screening and assessment completed *similarly—4th and 5th for multi-sector and public health, respectively. Those primarily working in health and acute care settings in the multi-sector group indicated that this indicator would be beneficial both as a proportion of older adults with a risk assessment and follow-up completed, but also with the ability to disaggregate risk assessments from follow up, along the continuum of care. This indicator could be used to take action to prevent an initial fall by knowing the number of older adults without a completed risk assessment, to secondary prevention of a subsequent fall for those at increased risk. There is a breadth of literature that supports risk assessment and follow up as an effective intervention for older adults [21]. Public health also placed this indicator 5th. This could be due to public health efforts to align their role as advocates for increasing reach and adoption of effective interventions in this population.

Another discrepancy between the groups was the ranking of the indicator *direct and indirect costs associated with falls*. The multi-sector group had this as their second highest ranked indicator, while it did not make it into the top 10 ranked indicators for public health. During discussions regarding this indicator, participants across sectors as well as specifically in the not-for-profit sectors expressed that being able to present the cost of a high-burden health issue to decision-makers, as is the case with falls, would prompt action and help make a case for investment in prevention programming. For those in public health, participants indicated that presenting the cost of falls often does not actually influence decision-makers to prioritize this work in practice, subsequently resulting in it being ranked lower.

Overall, the differing functions of public health compared to other health sectors was clearly reflected in each group’s respective priorities. While both groups prioritized indicators that capture the population burden of fall-related injuries, the public health group was more likely to prioritize indicators that report on the context and severity of falls and that would be useful for population-level intervention planning. Conversely, the multi-sector group was more likely to prioritize indicators that measure system performance. The indicators included in the top 10 were similar across both groups (8 the same, 2 different) their respective orders varied considerably, reflecting these different priorities. While foci differ between multi-sector and public health, together the two sets of indicators represent a whole system approach to older adult falls prevention, ranging from primary to tertiary prevention.

### Strengths and limitations

A strength of this work is the systematic and iterative process of gathering input from injury prevention practitioners in Ontario, ensuring that perspectives from each sector were represented at each stage and results being revised accordingly. Additionally, these methods were informed by previous work in this area [17–19], and experts in injury indicator development that provided feedback and guidance throughout. The results of this work are novel to Ontario, with many indicators being identified and specified for the first time in the province. Identifying the priority indicators for public health and across sectors in Ontario will help facilitate their systematic use and support consistent reporting over time and across sectors and regions.

There are, however, several limitations to this work. Firstly, due to the redeployment of many potential participants for this work to the COVID-19 pandemic response, participation rates were relatively low, with only 19 respondents to the survey. While we achieved participation from all relevant sectors, few had one representative and therefore may not have accurately reflected the thoughts of others in that sector. Additionally, during some feedback discussions, some sector representatives contributed disproportionately; as a result, we completed a pairwise comparison activity in an effort to ensure equal representation from each sector with equal weighting from each respondent. Finally, there are limitations to using the mDelphi approach itself. This includes the length of time needed from participants, no validated quality parameters to evaluate Delphi studies, no set protocol design nor consistent definition of consensus [22].

### Implications for practice

This work clearly identifies the fall prevention indicator priorities for public health, as well as across health sectors in Ontario. Our list of prioritized indicators align with previous work in this area [23]. Oakey et al. (2022) completed a similar process of ranking indicators based on importance and actionability with key decision-makers in British Columbia, Canada [23]. Eight indicators were prioritized for fall prevention including the top three related to burden (mortality, hospitalizations and ED visits), direct and indirect costs, wait time for surgery, health service coverage (which includes falls risk screening assessment, strength and balance exercise program, and home risk assessment coverage), dedicated fall prevention staff and the availability of fall prevention resources and plans [23]. The fall prevention indicators prioritized by decision-makers in British Columbia echo those prioritized in Ontario and strengthen our understanding of the intelligence required from data of those working in fall prevention across provinces. Identifying and prioritizing these indicators responds to the needs expressed by injury prevention practitioners for indicators that better reflect their work in fall prevention; however, indicators prioritized by this group should be considered within the list of indicators prioritized across sectors. This accounts for the reality that all systems have specialist areas, and specialist work foci within them; with emphasis that the system works most efficiently and effectively when all acknowledge and work within the system, rather than beside each other in unique (albeit complementary) systems. This will support the systematic use of indicators for reporting on fall-related injuries over time and across geographic regions, and to inform fall-prevention program development, implementation and evaluation.

Further, the differing priorities between sectors highlights the need for indicators that are useful for both and the importance of consulting practitioners when setting priorities. This work will support movement toward a system that centralizes data and indicator reporting in a consistent and systematic way and fulfills the actual needs of its end users.

## Conclusions

This work was successful in identifying and prioritizing indicators for older adult fall prevention across health sectors and for public health in Ontario. While both groups rank ordered indicators differently according to their differing foci and responsibilities in older adult fall prevention, together they reflect a whole system approach to falls prevention across all settings and sectors. Delineating the priority data and information needs of each group highlights the importance of having consistent and actionable indicators across the system.

The next step of this project is to populate the highest ranked indicators for public health, *the rate of falls by place of occurrence and activity* with region-specific data and pilot it among a subset of PHUs in Ontario. We will support participating PHUs with its use and seek to gather feedback on implementing the indicator in practice.

## Supplementary Information

Supplementary Material 1.

## Data Availability

The datasets used and/or analyzed during the current study available from the corresponding author on reasonable request.
